# Comparison of Diagnostic Yield and Complications in Ultrasound-Guided Closed Pleural Biopsy Versus Thoracoscopic Pleural Biopsy in Undiagnosed Exudative Pleural Effusion

**DOI:** 10.7759/cureus.23809

**Published:** 2022-04-04

**Authors:** Gopal Durgeshwar, Prasanta R Mohapatra, Shakti k Bal, Pritinanda Mishra, Sourin Bhuniya, Manoj K Panigrahi, Vedala Raja M Acharyulu, Sudip Ghosh, Satya P Mantha, Ananda Dutta

**Affiliations:** 1 Pulmonary Medicine and Critical Care, All India Institute of Medical Sciences, Bhubaneswar, Bhubaneswar, IND; 2 Pathology, All India Institute of Medical Sciences, Bhubaneswar, Bhubaneswar, IND

**Keywords:** diagnostic yield, pleural biopsy, ultrasound, medical thoracoscopy, exudative pleural effusion

## Abstract

Introduction

Malignancy, tuberculosis, and non-tubercular pleural infections account for most exudative pleural effusion. Pleural fluid cytology, biochemical tests and even pleural fluid cell block studies may fail to yield a diagnosis in certain cases. Medical thoracoscopy is the gold standard for the diagnosis of unexplained pleural effusions. However, access to medical thoracoscopy may be limited, particularly in developing countries. Also, certain patients may not be fit to undergo the procedure because of medical conditions. An ultrasound-guided pleural biopsy is an option in such conditions. The present study is intended to compare the diagnostic yield and complications of both methods of pleural biopsy in undiagnosed exudative pleural effusion under a randomized controlled trial.

Method

After fulfilling all the inclusion criteria, participants were randomized to either ultrasound-guided closed pleural biopsy or thoracoscopic-guided pleural biopsy groups. The primary outcome was to compare the diagnostic yield of ultrasound-guided Tru-Cut® (Newtech Medical Devices, Faridabad, India) closed pleural biopsy versus thoracoscopic pleural biopsy, and the secondary outcomes were to compare the complications rate, duration of the procedure, and hospital stay in the patients undergoing ultrasound-guided pleural biopsy versus thoracoscopic pleural biopsy, and predictors of a positive biopsy result in both groups.

Result

A total of 118 patients with pleural effusion were screened; 39 of them who were eligible, randomized into the ultrasound group (20 patients) and the thoracoscopic group (19 patients). The median age of participants was 53.5 (50-58) years and 55 (45-64) years in the ultrasound and thoracoscopic groups, respectively. Pleural fluid cell count, protein, adenosine deaminase (ADA), and lactate dehydrogenase (LDH) were similar in both groups, although pleural fluid glucose was low in the ultrasound group. Diagnostic yield was 90% (18/20) and 94.7% (18/19) in the ultrasound and thoracoscopic groups, respectively, which was statistically non-significant (p=0.963). The median duration of hospital stay was 9.5 (5.3-27) days and 15 (12-22) days in ultrasound and thoracoscopic groups respectively. The thoracoscopic group had a more prolonged stay compared to the ultrasound group, but it was statistically non-significant (p=0.09). The duration of the procedure was significantly longer in the thoracoscopic group 90 (85-105) minutes, in comparison to ultrasound 47.5 (41.3-55) minutes (p=0.001). No major complications were seen in both groups. Subcutaneous emphysema was the most common complication in the thoracoscopic group (10%), followed by hemorrhage (5.3%), and respiratory failure (5.3%). Hypotension was the only complication in the ultrasound group (5%). The rate of complications was significantly higher in the thoracoscopic group (p<0.01).

Conclusion

Ultrasound-guided closed pleural biopsy is as good as thoracoscopic pleural biopsy in undiagnosed exudative pleural effusion. It was associated with a shorter procedure duration, a shorter hospital stay, and fewer complications as compared to thoracoscopic biopsy. Both the procedures were safe in experienced hands and a hospital setup, but the thoracoscopic pleural biopsy was associated with complications.

## Introduction

Pleural effusion (PE) is an accumulation of excess fluid in the pleural space due to an imbalance between hydrostatic and oncotic pressure in the visceral and parietal pleural capillaries and persistent natural lymphatic drainage. The clinical diagnosis of pleural effusion is more straightforward than its etiological diagnosis. Finding an etiology is essential for management, as managements are different and maybe mutually exclusive. Common causes vary according to geographical location, disease endemicity, risk factors, and population demography [1,2]. Heart failure, pneumonia, and malignancies are the common causes in Europe and America, whereas tuberculosis (TB) is the most common cause in India and other developing countries [3,4].

A precise diagnostic workup can be done in 60-80% of patients after initial pleural fluid analysis, including biochemical (protein, glucose, adenosine deaminase, lactate dehydrogenase (LDH), and triglycerides), microbiological (routine gram staining and culture and mycobacterial culture), and cytological examination for atypical cells. However, after initial pleural fluid analysis, 20-40% of pleural effusion remains undiagnosed. This necessitates a pleural biopsy to establish the diagnosis [1,5]. Parietal pleural biopsy can be obtained via invasive procedures under direct vision (medical/surgical thoracoscopy) or semi-invasive techniques with or without radiologic image guidance (closed pleural biopsy (CPB)). Closed pleural biopsy (CPB) can be guided by computed tomography (CT), ultrasound (USG), or blind [6,7]. Image-guided pleural biopsy can be performed under ultrasound or computed tomography guidance by using a closed or cutting needle biopsy. Comparing CT-guided Tru-Cut® (Newtech Medical Devices, Faridabad, India) biopsy with closed-blind pleural biopsy, the diagnostic outcome (pathological) of CT-guided biopsy was 87% versus 47% for closed-blind pleural biopsy, suggesting Tru-Cut® needle biopsy nearly added 40% sensitivity in diagnosing malignancy (87% versus 47%) [8]. The sensitivity of medical thoracoscopy (MT) for the diagnosis of malignant pleural effusion ranges from 91% to 94%, while in tubercular pleural effusion it ranges from 93% to 100%.

Medical thoracoscopy procedures have some disadvantages, like the requirement of lateral decubitus position for the patient for at least 30 minutes, unbearable pain, or intractable cough, which may render the procedure unacceptable due to the risk of movement. Medical thoracoscopy is not well tolerated in patients with lung illness who cannot maintain optimal saturation when supine, and adequate oxygen saturation is required during conscious sedation and procedure. Supplemental oxygen may be necessary. Unfortunately, some patients are too frail to withstand medical thoracoscopy. Although this approach has risks [9], iatrogenic pneumothorax can be created in patients with minimal effusions to allow medical thoracoscopy to proceed. So, in a developing country where access to MT is limited, the patient presented with an advanced stage of malignancy, some time to frail and requiring supplement oxygen, requiring hospital admission and prolonged stay, and more post-procedure complications. Medical thoracoscopy is not always possible, so in this condition, USG-guided pleural biopsy is a better option with good diagnostic yield and fewer complications. Randomized prospective trials comparing USG-guided cutting needle biopsies and medical thoracoscopic pleural biopsies are occasionally available in the literature, and no Indian study is available to date.

## Materials and methods

This was a single-center, open-label, randomized control trial conducted in the Department of Pulmonary Medicine of our institute. Participants were enrolled in the study only after obtaining written informed consent. Patients aged ≥18 years with undiagnosed exudative pleural effusion were randomized to either ultrasound-guided closed pleural biopsy or thoracoscopic-guided pleural biopsy groups. Block randomization was done through a computer-generated program. Allocation was done by an opaque sealed envelope. However, participants and clinicians were not blinded to the procedure. Prior to randomization, baseline investigations were done, including complete blood count, renal function test, liver function test, prothrombin time, activated partial thromboplastin time, electrocardiogram, screening of viral markers (HIV, HbsAg, and hepatitis C virus (HCV)), pleural fluid analysis (glucose, protein, lactate dehydrogenase, adenosine deaminase, gram’s stain, culture and sensitivity, and pleural fluid cytology for malignant cell), and contrast-enhanced computed tomography (CECT) thorax. Patients with an age of >80 years or less than 18 years, central airway obstruction, bleeding diathesis/coagulopathy, extensive intrapleural inoculation, respiratory failure, severe comorbidities like recent myocardial infarction (within four weeks), acute and chronic renal failure, uncontrolled diabetes mellitus, pregnant women, patients on ventilator support, and hemodynamically unstable patients were excluded from the study.

Pleural biopsy (USG-guided and medical thoracoscopy)

**Figure 1:**
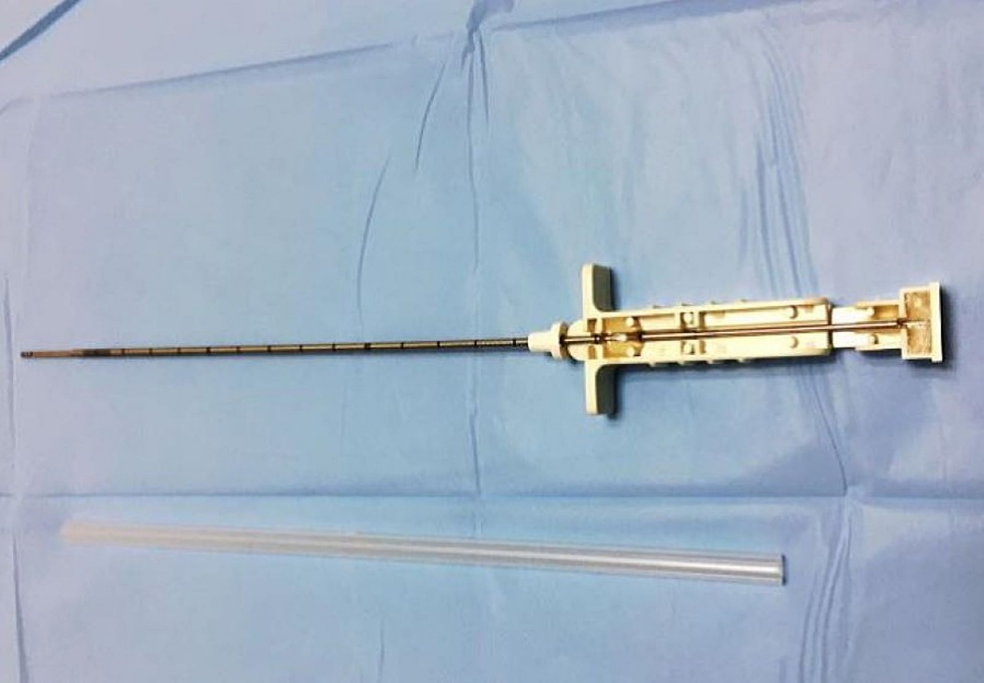
Tru-Cut® cutting biopsy needle.

**Figure 2:**
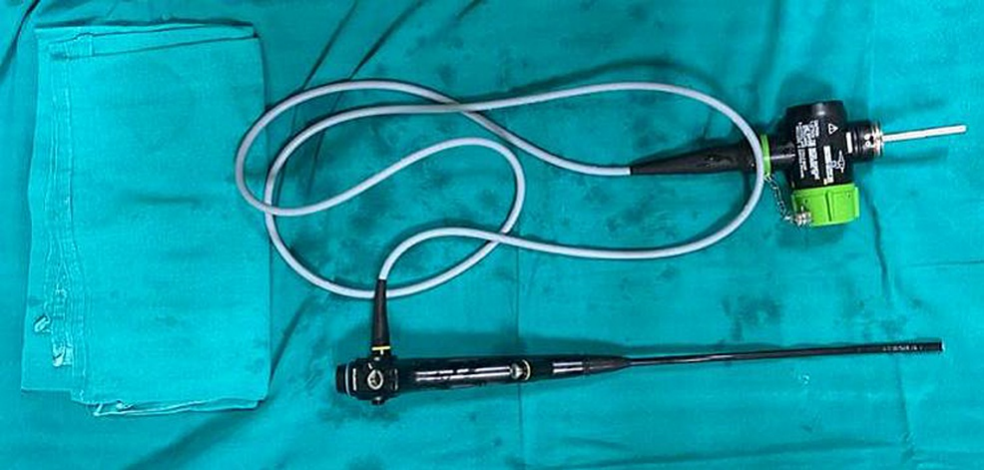
Olympus semi-rigid thoracoscope.

After a two-hour fast, localization of the site of the closed pleural biopsy was done by ultrasound chest (phased-array probe, 5-1 MHz). Areas of pleural thickening more than 25 mm were preferred for biopsy. Utmost care was taken to pursue a site of entry that is at least 6 cm away from the spinous process of the vertebra and on the upper order of the lower rib. A maximum of two sites for closed pleural biopsy were chosen. Two percent lignocaine was infiltrated into the skin, subcutaneous tissues, rib, periosteum, and parietal pleura. The pleura was punctured with the needle, and the site of entry was confirmed by aspiration of the free-flowing pleural fluid. A 2-mm incision was taken with an 11-size surgical blade and the 16-gauge or 14-gauge manual Tru-Cut® biopsy needle (Figure 1) was inserted. The correct site of entry was confirmed by hitting the upper border of the lower rib with the biopsy needle at every attempt of the biopsy. Furthermore, under real-time USG-guidance, the manually operated Tru-Cut® biopsy needle was advanced till the parietal pleura was reached. At this point, the Tru-Cut® needle was actuated to launch the specimen notch forward. The Tru-Cut® needle was withdrawn and the sample was dismantled from the specimen notch. USG and color doppler of the biopsy site(s) were performed after the procedure to exclude any pneumothorax or bleeding. Complications were treated according to standard protocol. Medical thoracoscopy was performed utilizing the Olympus, LTF‑Type 160 semi-rigid thoracoscope (Olympus Medical System Corporation, Tokyo, Japan) (Figure 2). The scope has a 22 cm proximal rigid insertion shaft with a 5 cm flexible tip and a 2.8 mm internal working channel for the introduction of forceps and other accessories. The high-resolution video imaging system provided sharp, clear images. While the patient was lying in the lateral decubitus position with the affected side up, the site of entry was marked with USG, about three intercostal spaces above the lower end of the effusion between the mid and posterior axillary lines, at a point above the upper border of the lower rib. In the aforementioned position, this usually corresponds to the fifth to seventh intercostal space. Intravenous access was obtained in the arm opposite to the site of the lesion. After a period of at least four hours of fasting, intramuscular pentazocine 50 mg and promethazine 25 mg were given. All the patients were connected to a pulse oximeter, blood pressure monitor, and nasal prongs with oxygen at a flow rate sufficient to maintain saturation above 95%. The patient was put in the same position as during the ultrasound-guided marking. At the marked site, two percent lignocaine was infiltrated into the skin, subcutaneous tissues, rib, periosteum, and parietal pleura. The pleura was punctured with the needle, and the site of entry was confirmed. Aseptic measures, a 1.5 to 2 cm skin incision parallel to the rib was given at the marked site. Thereafter, blunt dissection of the subcutaneous tissue was done with straight artery forceps till the pleura was punctured. At this point, the trocar and cannula were inserted. The trocar was removed and the pleural fluid was drained out with a 16-French suction catheter (Poly Medicure Ltd., Jaipur, India). After drainage of the pleural fluid, the thoracoscope was introduced, and an inspection of the pleura along with a video recording was done. Thereafter, biopsy forceps were introduced and biopsy pieces were obtained from areas of pleural irregularity. A 24-French intercostal drainage tube (ICDT) (Poly Medicure Ltd., Jaipur, India) was inserted and the cannula was removed over the ICDT. Thereafter, the ICDT was sutured to the skin and the site was covered aseptically with gauze pieces and a sticky bandage. Complications were treated according to standard protocol.

The primary outcome was to compare the diagnostic yield of ultrasound-guided Tru-Cut® closed pleural biopsy with that of thoracoscopic pleural biopsy. The secondary outcomes were to compare the complications rate, duration of the procedure, and hospital stay in patients undergoing ultrasound-guided pleural biopsy versus thoracoscopic pleural biopsy, and predictors of a positive biopsy result in both groups.

All statistical analysis was done using Epi Info Version 7.2.1.0 Statistical Software (Centers for Disease Control and Prevention (CDC), Atlanta, USA). Categorical/nominal variables were summarized as numbers and percentages and were analyzed using the Chi-square test or Fischer exact test as applicable, while continuous variables were expressed as median and interquartile ranges and were analyzed using the Mann-Whitney U test for comparison between the two groups. A p-value ≤0.05 was taken as statistically significant.

## Results

One hundred eighteen patients with pleural effusion were screened, out of which 39 patients were eligible for inclusion in the study. Participants were randomized to an ultrasound-guided pleural biopsy arm (20 patients, group A) and a thoracoscopic pleural biopsy arm (19 patients, group B). The male to female ratio in group A was 2:3, whereas in group B was 6:13. The median age of participants was 53.5 years (range 50-58 years) and 55 years (range 45-64 years) in groups A and B, respectively. Baseline characteristics of the patients in both groups are mentioned in Table 1.

**Table 1:** Baseline characteristics of the study population.

Parameters	Ultrasound group (n=20)	Thoracoscopic group (n=19)	P-value
Age (median in year)	53.5 (range 50–58 year)	55 (range 45–64 year)	0.922
Gender			0.83
Female	12 (60%)	13 (68.4%)	
Male	8 (40%)	6 (31.6%)	
Smoking status			0,90
Smokers	6 (30%)	7 (36.8%)	
Non-smokers	14 (70)	12 (63.2)	
Co-morbidities			
Diabetes mellitus	0 (0)	1 (5.3%)	1
Hypertension	4 (14%)	2 (10.5%)	0.60
Ischemic heart disease	0	0	

Baseline pleural fluid analysis (biochemistry and cell count) is mentioned in Table 2. Median cell count was 925/μl (range 481-1701.8/μl) and 960/μl (range 375-1735/μl), median ADA was 26.5 u/l (range 14-41.3 u/l) and 32 u/l (range 17.2-48 u/l), median protein was 4.8 g/dl (range 4.4-5.2 g/dl) and 5.2 g/dl (range 4.6-5.8 g/dl), and median LDH was 303 u/l (range 187-552.3 u/l) and 283 u/l (range 249- 361 u/l) in group A and group B, respectively. All the parameters were similar in both groups, except pleural fluid glucose, which was significantly lower in group B as mentioned in Table 2.

**Table 2:** Pleural fluid characteristics of study population. USG: ultrasound-guided, ADA: adenosine deaminase, LDH: lactate dehydrogenase, L: lymphocytes, N: neutrophils, and *significant.

Parameters	USG (n=20)	Thoracoscopy (n=19)	P-value
Glucose (median in mg/dl)	79.5 (range 47–97.3 mg/dl)	106 (range 78–135 mg/dl)	0.019*
ADA (median in u/l)	26.5 (range 14–41.3 u/l)	32 (range 17.2–48 u/l)	0.546
LDH (median in u/l)	303 (range 187–552.3 u/l)	283 (range 249–361 u/l)	0.747
Cells (median in µl)	925 (range 481–1701.8 µl)	960 (range 375–1735 µl)	0.933
L (median in%)	83.5 (range 80–94.8%)	84 (range 80–88%)	0.910
N (median in%)	18.5 (range 5.3–20%)	16 (range 11–19%)	0.778
Protein (median in g/dl)	4.8 (range 4.4–5.2 g/dl)	5.2 (range 4.6–5.8 g/dl)	0.078

One (2.6%) patient had bilateral pleural effusion, whereas 12 (30.8%) had left-sided pleural effusion and the other 26 (66.7%) had right-sided pleural effusion. No significant difference was seen in the side of pleural effusion between the two groups. Chest tomography (CT) of the thorax of the study population showed lung mass in 11 (28%) (six in group A, five in group B), pleural thickening in 17 (43%) (11 in group A, six in group B) and a pleural nodule in 4 (10%) (all in group B). The majority of the patients in the USG arm had a pleural thickness of more than 0.5 cm. In the case of non-uniform pleural thickening maximum, the pleural thickness was taken (like if the pleural thickness was from 1.2 to 1.4, then 1.4 cm was taken).

In the thoracoscopic arm, 68% (13/19) had pleural nodules, 52% (10/19) had diaphragmatic nodules, 47% (9/19) had pleural adhesion, 31% (6/19) had parietal pleural infiltration, 26% (5/19) had visceral pleural infiltration, and 10.5% (2/19) had normal pleura on visual inspection of the pleural cavity during medical thoracoscopy. Out of thirteen, seven had grape-like nodules, five had sago grain nodules, and one had cauliflower-like nodules.

Diagnostic yield was 94.7% (18/19) and (18/20) 90% in the USG arm and thoracoscopic arm, respectively, which was statistically non-significant (p=0.963). Two patients remained undiagnosed even after an ultrasound-guided pleural biopsy, which according to the protocol, would be subjected to thoracoscopic pleural biopsy but could not be performed because of the COVID-19 pandemic. Pleural malignancy was the most common histopathological diagnosis in both groups (70% in group A, 52.6% in group B), followed by tubercular pleuritis (15% in group A, 36.8% in group B). Among malignancies, metastatic adenocarcinoma of the lung was the most common (40% in group A, 26.3% in group B), followed by pleural mesothelioma (10% in group A, 15.8% in group B). Three cases were diagnosed as small cell carcinoma of the lung (all in group A). One case was of metastatic adenocarcinoma from the breast in each group, and one case was of metastatic adenocarcinoma from an unknown primary in group B. A total of two patients were diagnosed with granulomatous pleuritis, one in each group. In the secondary outcome, the median duration of hospital stay was 9.5 days (5.3-27 days) and 15 days (12-22 days) in group A and group B, respectively. Group B had a more prolonged stay than group A but was statistically non-significant (p=0.09). The median duration of the procedure was 47.5 minutes (41.3-55 minutes) in group A and 90 minutes (85-105 minutes) in group B. The duration of the procedure was significantly longer in group B compared to group A (p=0.001). Subcutaneous emphysema was the most common complication in group B (10%), followed by bleeding (5.3%), and respiratory failure (5.3%), with significantly more complications observed in group B (p < 0.01).

## Discussion

This study found that ultrasound-guided closed pleural biopsy is as effective as medical thoracoscopic pleural biopsy in diagnosing unexplained exudative pleural effusion. The diagnostic yield was slightly higher in the thoracoscopy arm but was statistically non-significant. The duration of the procedure was significantly longer in the thoracoscopic arm compared to the ultrasound arm. The hospital stay was also longer in the medical thoracoscopic arm than in the ultrasound-guided closed pleural biopsy arm, but it was statistically non-significant. The pleural biopsy method is safe in experienced hands but associated with minor complications, with slightly higher complications in the thoracoscopy arm.

According to the British Thoracic Society 2010 pleural disease guideline [1], thoracoscopy is the gold standard procedure for diagnosing unexplained exudative pleural effusion. Direct visualization of the pleural space, drainage of pleural fluid, and performing therapeutic intervention in the same session are advantages of medical thoracoscopy. The disadvantages include limited accessibility (mainly in a developing country), the requirement of supplement oxygen, hospitalization, prolonged stay, and post-procedure complications.

This study aimed to compare the diagnostic yield, hospital stay, and complications of thoracoscopic and ultrasound-guided pleural biopsy. Randomized prospective trials comparing USG-guided cutting needle biopsies and medical thoracoscopic pleural biopsies, head-to-head, are occasionally available in the medical literature. However, there is no Indian study published to date.

Our study was a randomized control trial that enrolled 39 patients with undiagnosed exudative pleural effusion. Medical thoracoscopy was performed in 19 patients, and ultrasound-guided pleural biopsy in 20 patients. In our study, the diagnostic yield of medical thoracoscopy was 94.7%, indicating a high yield in undiagnosed exudative pleural effusion, which is similar to the diagnostic yield of 90 to 97% reported in various studies [10-13]. A diagnostic yield of 66 to 97% has been reported in Indian studies [14-18]. A higher yield can be explained by the fact that thoracoscopy allows for better visualization of the pleural cavity, including the costal, diaphragmatic, and visceral pleural surfaces, as well as the underlying lung, providing more information on the extent of the disease and allows for real-time and adequate pleural tissue sampling from the diseased pleura.

Pleural malignancy and tubercular pleuritis are common causes of undiagnosed exudative pleural effusion. In our study, the most common diagnosis was pleural malignancy (62%), 52% in the thoracoscopic arm, and 70% in the ultrasound-guided pleural biopsy arm, in which adenocarcinoma of the lung was the most common primary malignancy. This is similar to the results reported by Prabhu et al. [18] by medical thoracoscopy but in contrast to Dhooria et al. [16] and Kannan et al. [19] in which tubercular pleuritis was the most common cause of undiagnosed pleural effusion by medical thoracoscopy. A similar result in the ultrasound arm was reported by Mansour et al. [20].

In our study, higher malignant pleural effusion in the TB endemic population can be explained by the fact that our department is a tertiary care center for diagnosing and managing lung cancer. In our country, anti-tubercular treatment (ATT) is often started in pleural effusion on a presumed clinical basis; therefore, most of the patients improve on ATT, so they are less likely to be referred, which at times leads to referral bias. Two patients who had granulomatosis pleuritis on each arm were started on antituberculosis treatment on a clinicoradiological basis, and both patients improved on treatment.

In our study, the diagnostic yield of ultrasound-guided pleural biopsy was 90%, which was similar to the study by Chang et al. [21] in which the diagnostic yield of ultrasound-guided Tru-Cut® pleural biopsy was 87%, and other studies with diagnostic yields ranging from 70 to 94% [20,22-24]. The higher diagnostic yield in our study is explained by the fact that ultrasound allows real-time imaging of the pleural space, helps to identify the entry site, a real-time image-guided pleural biopsy from the most thickened accessible parietal pleural, the use of a cutting biopsy needle, a larger size biopsy needle, the greater number of pleural biopsy samples obtained, and pleural thickening of more than 3 mm in the majority of the patients. These are the predictors consistent with higher diagnostic yield in ultrasound-guided pleural biopsy in a study done by Zhang et al. [22]. In the ultrasound arm, the higher diagnostic yield was seen in patients with a pleural thickness on CT and a pleural thickness of more than 3 mm on ultrasound, but this was statistically non-significant because of the small sample size.

There are a few randomized control trials suggesting that image-guided pleural biopsy is as effective as thoracoscopic pleural biopsy in undiagnosed pleural effusion. In our study, the diagnostic yield was 94.7% and 90% in the thoracoscopic arm and ultrasound arm, respectively, which is similar to the reported by Mohamed et al. [25] and Mansour et al. [20] compared image-guided pleural biopsy by Abram needle versus medical thoracoscopy.

Duration of the procedure

In our study, the median duration of the procedure in thoracoscopic pleural biopsy was longer than in ultrasound-guided pleural biopsy, a similar result found by Ibrahim et al. [26]. The longer procedural time observed in the thoracoscopic arm can be explained by the fact that it required blunt dissection followed by trocar insertion, a thorough inspection of the pleural cavity, selection of an appropriate biopsy site, intercostal tube drainage, and suturing.

Hospital stay

The length of hospital stay was calculated from the day of the procedure to the day of discharge. In our study, the hospital stay was longer in the thoracoscopic group than in the ultrasound group, but the difference was statistically non-significant. Several factors could have contributed to the longer hospital stays in our study in both groups. Firstly, in our tertiary care government hospital where inpatient care is delivered free of cost, including imaging and staging evaluation for cancer patients, many patients overstay in the hospital beyond clinical indication for staging, evaluation, related investigation and treatment planning, including palliative chemotherapies. In the thoracoscopic arm, patients with ICDT are usually reluctant to go home with the tube in situ, and they prefer tube removal before discharge due to a lack of an adequate social support system and trained personnel to take care of the ICDT-related issues at their residence. As such, the morbidity associated with the ICDT insertion generally prolongs the hospital stay. We used to get the histopathology reports, and patients needed to stay for palliative chemotherapy by the time we used to get the histopathology reports.

The majority of the patients in the ultrasound-guided pleural biopsy group had malignant etiology. Repeated therapeutic thoracentesis was required and, in a few patients, pleurodesis was performed, which led to a further hospital stay. The majority of the patients in our study had malignant pleural effusion. As a result, they came under advanced stages of lung cancer due to the involvement of the pleura (M1a), therefore becoming unresectable malignancy. The targeted treatment or palliative chemotherapy and pleurodesis remain available treatment options in our department. All these processes, like the pre-chemotherapy injection of vitamin B12 (cyanocobalamin) seven days before pemetrexed, even molecular tests take time, leading to a prolonged hospital stay in both arms. All these processes prolong the hospital stay.

Complication

Most studies documented major complications of medical thoracoscopy like empyema, hemothorax, pneumonia, tumor seeding along the procedure tract, pneumothorax, and persistent air leak. Minor complications included subcutaneous emphysema, minimal bleeding, local wound infection, hypotension during the procedure, and transient fever [27]. In our study, most of the procedure-related complications were minimal, and procedure-related mortality was not seen in both groups. Tolerance to the procedure was good in both groups, as no premature termination of the procedure was seen. The most common complication in the ultrasound group was intraprocedural pain. One patient in the ultrasound arm developed transient post-procedural hypotension, which was managed with intravenous fluids. No major complications were seen in the ultrasound arm like pneumothorax, hemothorax, and respiratory failure, a similar result found in a study done by Bahr et al. [28]. The procedural hypotension has also been documented in a study by Diacon et al. [29]. It is likely due to vasovagal syncope, which improved with the administration of intravenous fluid.

In the thoracoscopic arm, the most common complication was subcutaneous emphysema, followed by respiratory failure and empyema. These results are consistent with Blanc et al. [30] and Prabhu et al. [18]. One patient developed respiratory failure in the thoracoscopic arm, which was managed with oxygen. During hospital stays, one patient got infected with SARS-CoV-2 and suffered from mild COVID-19 and later developed empyema, which was managed with antibiotics and discharged in stable condition. So both the methods of pleural biopsy are safe in experienced hands, with slightly higher complications in the thoracoscopy arm.

To the best of our knowledge, this was the first Indian randomized control trial that compared the diagnostic yield and complications of thoracoscopic pleural biopsy and ultrasound-guided pleural biopsy in undiagnosed pleural effusion. We aimed to find out if ultrasound-guided pleural biopsy can be an alternative to thoracoscopic pleural biopsy in our country where access to medical thoracoscopy is limited and the patients present at an advanced stage of the disease with poor performance status, a situation where a more invasive procedure like a thoracoscopic biopsy is not always possible, leading to a delay in the diagnosis and poor outcome.

Our study has several limitations. The very nature of the interventions in both arms precluded any blinding. The sample size was small as we could not enroll the required number of participants in this trial because of the COVID-19 pandemic. The smaller sample size probably led to a statistically non-significant result.

## Conclusions

The ultrasound-guided closed pleural biopsy is as good as thoracoscopic pleural biopsy in undiagnosed exudative pleural effusion. Also, the ultrasound-guided pleural biopsy was associated with a shorter procedure duration, shorter hospital stay, and fewer complications as compared to thoracoscopic pleural biopsy. Both procedures were safe in the experienced hand and a hospital setup. Further trials, including a larger number of participants, are needed to confirm our findings.
